# Recovery of Isoamyl Alcohol by Graphene Oxide Immobilized Membrane and Air-Sparged Membrane Distillation

**DOI:** 10.3390/membranes14020049

**Published:** 2024-02-10

**Authors:** Mitun Chandra Bhoumick, Sumona Paul, Sagar Roy, Benjamin G. Harvey, Somenath Mitra

**Affiliations:** 1 Department of Chemistry and Environmental Science, New Jersey Institute of Technology, Newark, NJ 07102, USA; mb777@njit.edu (M.C.B.); sp2652@njit.edu (S.P.); sagar.roy@njit.edu (S.R.); 2 Naval Air Warfare Center, Weapons Division, Research Department, Chemistry Division, China Lake, CA 93555, USA; benjamin.g.harvey.civ@us.navy.mil

**Keywords:** isoamyl alcohol, graphene oxide, Box–Behnken design, separation factor, active sorption–desorption

## Abstract

Isoamyl alcohol is an important biomass fermentation product that can be used as a gasoline surrogate, jet fuel precursor, and platform molecule for the synthesis of fine chemicals and pharmaceuticals. This study reports on the use of graphene oxide immobilized membra (GOIMs) for the recovery of isoamyl alcohol from an aqueous matrix. The separation was performed using air-sparged membrane distillation (ASMD). In contrast to a conventional PTFE membrane, which exhibited minimal separation, preferential adsorption on graphene oxide within GOIMs resulted in highly selective isoamyl alcohol separation. The separation factor reached 6.7, along with a flux as high as 1.12 kg/m^2^ h. Notably, the overall mass transfer coefficients indicated improvements with a GOIM. Optimization via response surfaces showed curvature effects for the separation factor due to the interaction effects. An empirical model was generated based on regression equations to predict the flux and separation factor. This study demonstrates the potential of GOIMs and ASMD for the efficient recovery of higher alcohols from aqueous solutions, highlighting the practical applications of these techniques for the production of biofuels and bioproducts.

## 1. Introduction

Bioderived chemicals offer a viable alternative to conventional fossil fuels, providing an opportunity to reduce greenhouse gas emissions and decrease dependence on finite resources [1,2,3]. Notably, the automotive fuel sector is poised to see a rapid increase in the market share of biofuels over the next decade due to their environmental benefits. Bioderived chemicals and fuels have several advantages, including ready availability from common biomass sources, their role in the carbon dioxide cycle during combustion, significant environmentally friendly potential, various benefits for the environment, economy, and consumers, and their biodegradability, promoting sustainability [1,2]. It is important to note that a key distinction between biofuels and petroleum feedstocks lies in their oxygen content, with biofuels containing 10% to 45% oxygen, as opposed to essentially none in petroleum. Additionally, biofuels typically have very low sulfur and often low nitrogen levels [3,4].

Biofuels exist in various forms, each with unique production processes and applications in the renewable energy landscape [5,6]. One prominent category is bioethanol, which is commonly blended with gasoline to create ethanol blends for use in conventional vehicles. Biodiesel, another significant biofuel, is produced through the transesterification of vegetable oils or animal fats [7,8,9,10]. It serves as a renewable alternative to traditional diesel fuel and can be used in diesel engines with minimal to no modification [4,11,12,13,14]. Biogas, generated through the anaerobic digestion of organic materials like agricultural residues or wastewater, mainly consists of methane and carbon dioxide. This versatile fuel can be used for electricity generation, heating, or as a vehicle fuel. Advanced biofuels are produced from non-food biomass sources like agricultural residues, wood, or dedicated energy crops [12,15,16]. Algae-based biofuels leverage photosynthetic microorganisms to convert sunlight into energy-rich compounds, offering a potential alternative to traditional biofuel feedstocks. The diversity of biofuels reflects ongoing efforts to explore sustainable alternatives and reduce dependence on fossil fuels, contributing to a more environmentally friendly and diversified energy portfolio [10,17,18].

The production of bioderived alcohols aligns with the principles of a circular bioeconomy, utilizing renewable resources to generate energy and reduce reliance on finite fossil fuels [19,20,21]. One primary advantage of bioderived alcohols is their potential to significantly lower greenhouse gas emissions compared to traditional fossil fuels when used as transportation fuels [22,23,24,25]. Furthermore, their use contributes to agricultural diversification, providing economic benefits to farmers and rural communities [9,26,27,28,29]. Ongoing research and advancements in biotechnology aim to enhance the efficiency and sustainability of bioderived alcohol production, further solidifying their role as a cleaner, greener alternative in the global energy landscape [6,8,30].

Several studies have delved into the utilization of isoamyl alcohol as a viable substitute for ethanol in compression engines, and the outcomes have shown great promise [31,32,33].

Isoamyl alcohol has also been explored as a precursor to aviation fuels. For example, a recent study described the conversion of fusel alcohols, including isoamyl alcohol, into potential jet fuel surrogates through Lewis acid catalyzed dehydration and oligomerization [31,34]. Similar approaches, which leverage bioderived branched chain C5 platform chemicals, are currently being exploited for the production of next-generation sustainable aviation fuels [35]. Beyond fuels, isoamyl alcohol serves as a platform chemical for the synthesis of isopentyl acetate, resins, oils, natural flavors, cellulose acetate, pharmaceuticals, and pesticides. Finally, isoamyl alcohol is employed in DNA extraction, often in conjunction with phenol and chloroform, functioning as an anti-foaming agent [36,37].

Isoamyl alcohol can be produced through various methods, including chemical, and biological processes [4,38,39]. The fermentation of carbohydrates using specific strains of bacteria or yeast is an example of the latter. The concentration of biosynthetic isoamyl alcohol typically ranges between 2 and 5% in the fermentation broth. This modest concentration inhibits the ability to isolate isoamyl alcohol and efficiently convert it to value-added derivatives [31,40,41].

Various methods are employed for the recovery of isoamyl alcohol, including distillation, adsorption, and solvent extraction. Distillation is commonly utilized to separate isoamyl alcohol from other alcohols and impurities based on differences in their boiling points [25,33,42,43]. Adsorption and solvent extraction techniques have been used. The selection of a specific recovery method depends on factors such as the desired purity of the product, the required quantity, and the cost-effectiveness of the method [44,45,46]. In general, the aforementioned methods tend to be expensive and/or energy intensive. Therefore, the development of alternative recovery processes is important to ensure the efficient and economically viable production of isoamyl alcohol.

Membrane distillation (MD) presents a promising alternative method for concentrating isoamyl alcohol from fermentation broth. MD utilizes a hydrophobic membrane to segregate volatile compounds from a liquid stream by exploiting disparities in vapor pressures [47,48,49]. The vapor pressure difference is achieved by establishing a temperature gradient across the membrane [1,2]. The volatile components produced on the hot side permeate through the membrane and condense on the cold side, where they are collected as a purified product. In this procedure, a hydrophobic membrane facilitates the passage of vapor while impeding the movement of liquid, preventing wetting and fouling for an effective separation process [50,51,52]. The selective permeation of the hydrophobic membrane ensures the rejection of impurities, yielding a relatively pure distillate on the cold side.

There are various types of membrane distillation (MD), each employing distinct configurations and operational principles for the separation of vapor from liquid [53,54,55]. Direct contact membrane distillation (DCMD) involves a hydrophobic membrane that separates hot and cold streams, with heat applied to the feedwater, increasing its vapor pressure [56,57,58]. Air gap membrane distillation (AGMD) introduces an air gap between the membrane and the hot liquid stream, where water vapor passes through the membrane into the air gap [59,60,61]. Vacuum membrane distillation (VMD) operates under reduced pressure, useful for heat-sensitive compounds. Sweep gas membrane distillation (SGMD) employs a sweep gas on the cold side to carry away water vapor [62,63,64,65].

Recently, we have reported the development of air-sparged membrane distillation (ASMD) for the treatment of saline water and solvent recovery, where air sparging helps to reduce membrane fouling and increase flux [66]. Air sparging has also shown significant enhancement in the separation factor for the volatile components. Another development in MD technology is the utilization of nanocarbon immobilized membranes which show a better performance in terms of flux and selectivity [49,67]. Our group has shown the application of carbon nanotube and graphene oxide modified membranes for the separation of water and solvents. In particular, graphene oxide immobilized membranes (GOIM) offer a number of advantages due to their functionality and selectivity for organic solvents [68,69,70].

The objective of this study was to investigate the application of ASMD for the recovery and concentration of isoamyl alcohol from a dilute aqueous stream. Air sparging was used on the feed side to enhance the volatilization of isoamyl alcohol. In this study, ASMD is selected in an SGMD configuration because of the ease of product collection. Yet another objective was to study the effectiveness of the GOIM for the separation via MD.

## 2. Experimental

### 2.1. Chemicals and Materials

Isoamyl alcohol (TCI America, Portland, OR, USA) and acetone (Fisher Scientific, Fair Lawn, NJ, USA), and PTFE membranes (AdvantecTokyo, Japan), were used without further modification. In all experiments, deionized water was used. Graphene oxide (GO) was purchased from Cheap Tubes Inc., Brattleboro, VT, USA.

### 2.2. Membrane Fabrication

The fabrication of graphene oxide membranes (GOIM) was carried out using a two-step process. In the first step, GO was uniformly dispersed in acetone using ultrasonication, following a methodology reported elsewhere [66]. For each membrane, 1.5 mg of GO was dispersed in 10 mL of acetone. In a separate vial, 0.2 mg of PVDF was mixed with 2 mL of acetone and sonicated to obtain a homogeneous solution, which was then added to the dispersed nanomaterials as a binder during the immobilization process. The membrane surface was drop coated with the dispersed GO suspension and left to dry overnight under a hood at room temperature. A polytetrafluoroethylene laminate backed on a polypropylene composite membrane was used as a substrate for GO dispersion.

### 2.3. Experimental Set-Up

**Figure 1:**
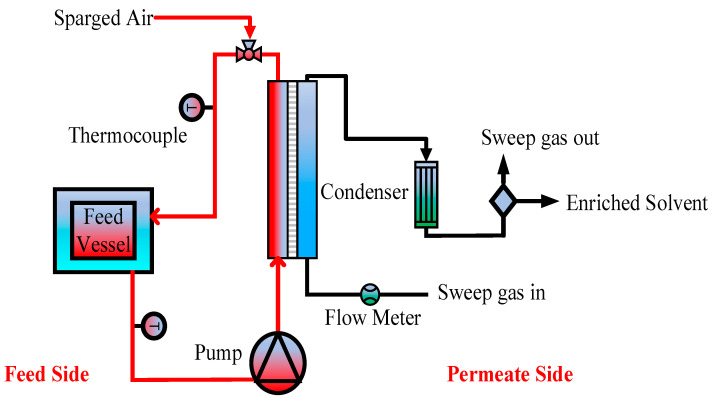
Experimental setup for isoamyl alcohol recovery via AS-SGMD.

The experiments were run in a bench-scale MD system. The setup for air-sparged sweep gas membrane distillation (AS-SGMD) is shown in Figure 1. The membrane module was made of polytetrafluoroethylene. In the experimental setup, the hot feed entered the module’s feed side, while a cool gas efficiently swept through the permeate side to eliminate the generated vapor. To minimize heat loss, insulated tubing was strategically employed. The experiments utilized an effective membrane surface area of 11.80 cm^2^. The feed inlet diameter was 1 cm. The module was arranged vertically. Circulation of the hot feed was facilitated by a peristaltic pump (Cole Parmer, Vernon Hills, IL, USA, model 77200-52), its temperature meticulously regulated by a constant temperature water bath. K-type temperature sensors ensured precise monitoring of the feed and permeate temperatures. Dry laboratory air functioned as the sweeping gas, and the flow rates of the sparged air and permeate-side air were meticulously maintained at around 0.25 L/min and 2 L/min, respectively, measured using digital flow meters (Kelly Pneumatics, Inc., Newport beach, CA, USA). The relative humidity of the supplied air was below 20%. The permeate was collected using a cold trap. The experiments, conducted in triplicate, exhibit a reported relative standard deviation, emphasizing the reliability of the findings.

A digital lab scale was used to measure changes in feed weight. To avoid any unintended loss of feed during the experiment, we employed a tightly sealed feed chamber. The composition of the feed mixture before and after the experiment was analyzed using a gas chromatograph (HP-5890, Wilmington, DE, USA) with a flame ionization detector (FID). We separated components on a Restek capillary column with dimensions (30 m × 0.32 mm ID × 1 μm). Helium served as the carrier gas, flowing steadily at 1 mL/min. Injections were made with a split ratio of 20:1, and the injector temperature was held at 200 °C. The initial temperature of the gas chromatography (GC) oven was set to 55 °C, maintained for 10 min, then gradually increased to 200 °C at a rate of 25 °C per minute, and finally held for an additional 10 min. These procedures ensured accurate measurements and reliable analysis throughout the experiment.

### 2.4. Flux and Separation Factor in Membrane Distillation

In membrane distillation, flux is defined as the rate at which water vapor permeates through the membrane per unit area. This flux directly affects the overall performance of membrane distillation systems. Several factors influence flux, including the temperature difference between the hot and cold sides of the membrane, membrane properties such as thickness and hydrophobicity, the characteristics of the feed solution such as temperature and concentration, the transmembrane pressure difference, and the occurrence of membrane wetting. In this study, the isoamyl alcohol flux across the membrane is expressed as follows.

(1) $$J_{\mathrm{Isoamyl}\;\mathrm{Alcohol}}=\frac{W_{\mathrm{Isoamyl}\;\mathrm{Alcohol}\;}}{t\times A}$$

where W_isoamyl_ alcohol is the amount of isoamyl alcohol passed through the membrane at time t, and A is the effective membrane area exposed to sorption in the separation process.

Another important MD performance parameter is the separation factor; this refers to the ability of the membrane to preferentially allow specific components to permeate through while inhibiting the passage of other substances in the feed solution. It is a critical parameter that quantifies the membrane’s efficiency in separating and purifying the desired component. The selectivity of a membrane is typically assessed by comparing the concentration of a specific substance in the permeate to its concentration in the feed solution. A higher separation factor indicates a greater ability to discriminate in favor of the target component, contributing to the overall effectiveness of the membrane distillation process, particularly in applications such as desalination, solvent enrichment, and purification. In this study, both water and isoamyl alcohol pass through the membrane as vapor. The preferential transport of isoamyl alcohol is desired for a high separation factor. The amount of isoamyl alcohol permeating through the membrane was calculated from the measured isoamyl alcohol concentration in the permeate and the weight of the collected permeate. The efficiency of separation for each membrane studied was represented by separation factor (α_i−j_) and is estimated by the following mathematical relation.

(2) $$\alpha_{\mathrm{isoamyl}\;\mathrm{alcohol}-\mathrm{water}}=\left(\frac{y_{\mathrm{Isoamyl}\;\mathrm{Alcohol}}}{y_{\mathrm{water}}}\right)/\left(\frac{x_{\mathrm{Isoamyl}\;\mathrm{Alcohol}}}{x_{\mathrm{water}}}\right)$$

where y and x represent the permeate and feed-side weight fraction of the corresponding component.

## 3. Membrane Characterization

**Figure 2:**
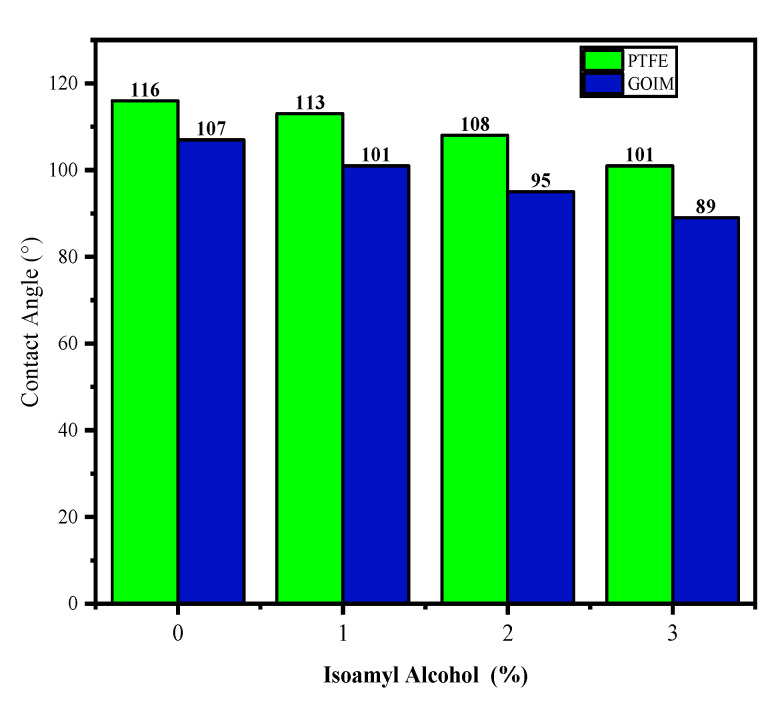
Contact angle of the membranes for various isoamyl alcohol concentrations. The concentration ranged from 0 to 3 *w*/*w*% in water. The GO loading was held constant at 0.10 mg/cm^2^.

The characterization of the membrane surface was meticulously conducted utilizing scanning electron microscopy (SEM) with a JEOL JSM 7900 (Oxford Instruments, Tokyo, Japan). This technique provided detailed insights into the topographical features and morphology of the membrane. To assess the thermal stability of the membranes, comprehensive thermogravimetric analysis (TGA) was performed, employing a Perkin Elmer 8000 apparatus (Shelton, CT, USA). This analysis involved subjecting the membranes to varying temperatures to study their weight changes over time, offering valuable information about their thermal stability. The study delved into the dynamics of liquid–membrane interactions through contact angle measurements. Aqueous solutions of isoamyl alcohol with diverse concentrations in deionized water were utilized for this purpose. Figure 2 shows the contact angles for distilled water and aqueous isoamyl alcohol solutions on the membrane surface. The incorporation of graphene oxide (GO) into the membrane induced significant alterations in the contact angles when compared to the control membrane. The specific values of these contact angles are elucidated in Figure 2, providing a nuanced understanding of the surface interactions. Furthermore, the data presented in Figure 2 highlight that GO exhibited a pronounced affinity and interaction with isoamyl alcohol, showcasing its distinctive influence on liquid–membrane dynamics as opposed to the control membrane. This comprehensive analysis contributes valuable insights into the intricate interplay between membrane composition and its interactions. This is consistent with our previous studies where we observed enhanced interactions with ethanol and other organics [3,4,5].

**Figure 3:**
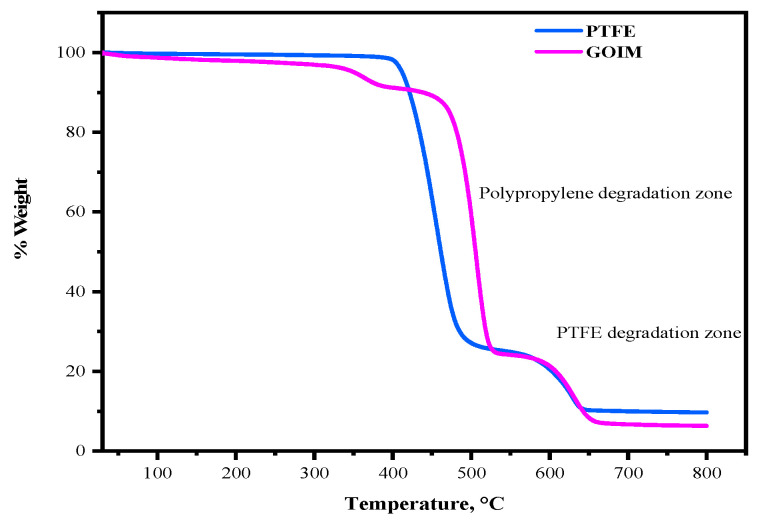
Thermogravimetric analysis of the control PTFE and GOIM membranes.

Figure 3 illustrates the thermogravimetric analysis (TGA) curves for both polytetrafluoroethylene (PTFE) and graphene-oxide-modified PTFE (GOIM) membranes. The decomposition of polypropylene initiated a weight loss of the membranes at 350 °C, with a significant weight change occurring within the temperature range of 450 to 500 °C. The deterioration of PTFE was evident in the temperature span of 550 to 600 °C. However, in the presence of GO in the modified membranes, the GOIM exhibited an enhanced stability in the temperature range of 450 to 600 °C, demonstrating a lower degradation rate compared to the PTFE membrane. This observation underscores the positive influence of GO on the thermal stability of the membrane.

**Figure 4:**
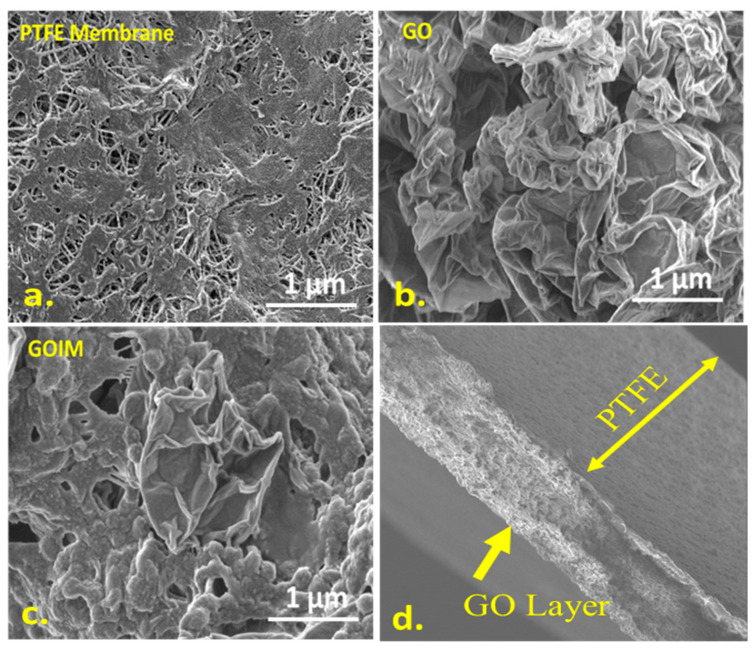
Scanning electron microscopic images of the membranes; (**a**) unmodified PTFE membrane, (**b**) graphene oxide, (**c**) graphene oxide immobilized membranes and (**d**) cross section of the GOIM membrane.

SEM images of the control PTFE membrane, graphene oxide nanoparticles, GOIM, and the cross section of the GOIM are shown in Figure 4a–d. The GOIM exhibits a wrinkled structure with dispersed GO particles on the surface. A layered GO SEM image is provided as a reference. The cross-sectional image in 4d clearly shows the surface deposition of the GO on the PTFE membrane.

The membrane porosity of both the PTFE and GOIM membranes was measured using a gravimetric method. The porosity of the PTFE membrane was 0.73; meanwhile, the porosity of the graphene oxide immobilized membrane was found to be 0.71. The low nanocarbon loading and porous nature of graphene oxide nanoparticles did not change the porosity of the modified membranes to a significant degree.

## 4. Results and Discussion

### 4.1. Antoine Equation for the Isoamyl Alcohol–Water System:

**Figure 5:**
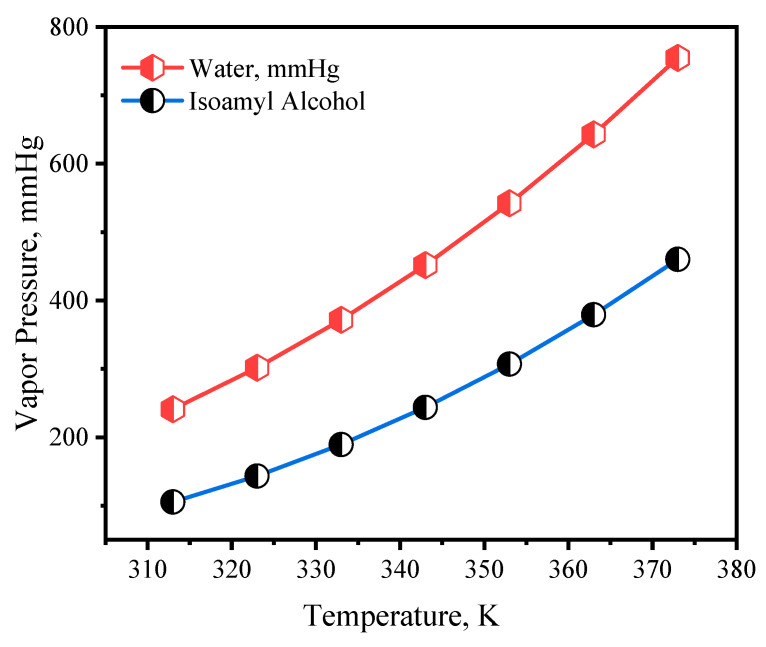
Vapor pressure of pure isoamyl alcohol and water using the Antoine equation.

The relationship between the isoamyl alcohol vapor pressure and temperature is shown in Figure 5 as determined using the Antoine equation in the following form.

lnP^s^ = A + B/(T + C) (3)

where A, B, and C are constants having values of 16.71, 3026.43, and −104.1, respectively. The applicable temperature range for this equation is 298–426 K. The vapor pressure of isoamyl alcohol increased exponentially with temperature. In a mixture of isoamyl alcohol and water, the isoamyl alcohol–isoamyl alcohol interaction is greater than that between isoamyl alcohol and water. According to Raoult’s law, this phenomenon could lead to a positive deviation in vapor pressure.

### 4.2. Effect of Operating Variables on Flux and Separation Factor

**Figure 6:**
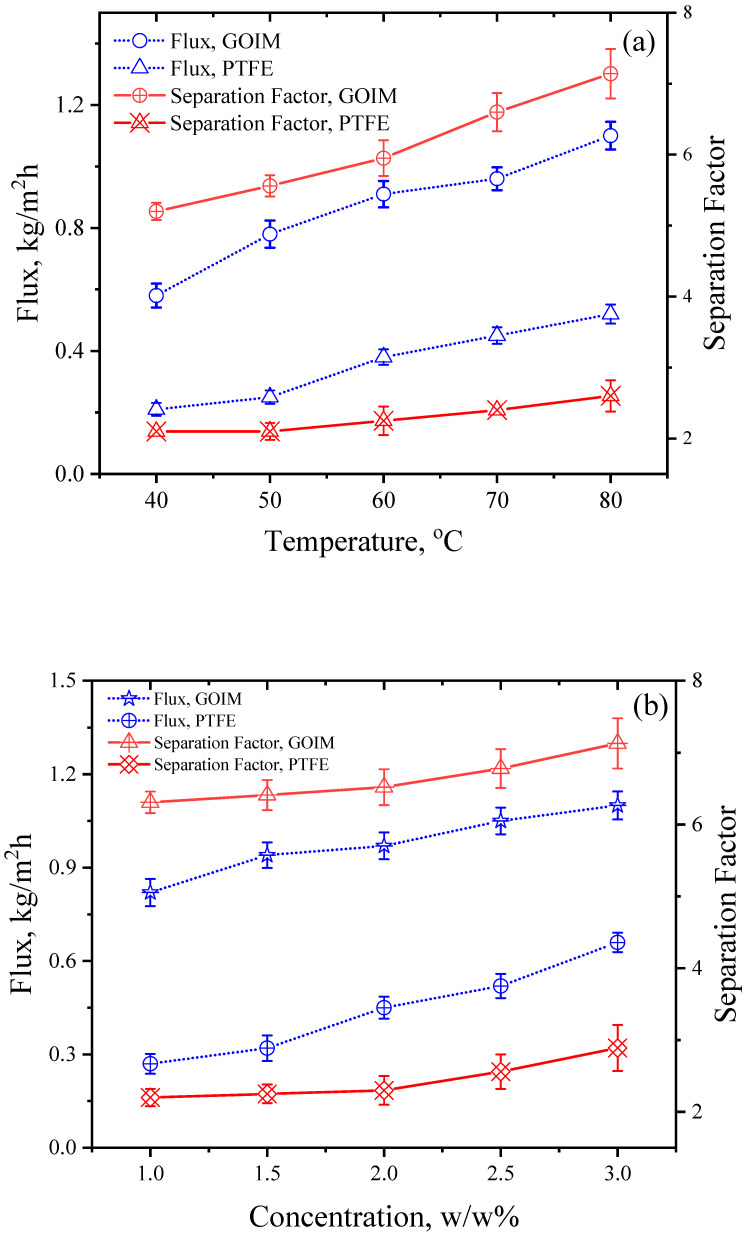

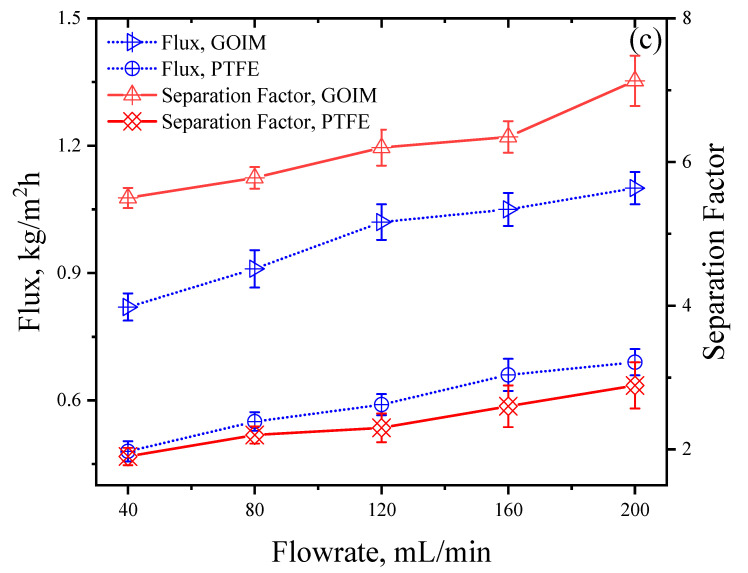
Flux and separation factor for isoamyl alcohol with respect to (**a**) Temperature (flow rate: 200 mL/min and concentration: 3 *w*/*w*% kept constant) (**b**) Concentration (temperature: 80 °C and flow rate: 200 mL/min kept constant) (**c**) Flow rate (temperature: 80 °C and concentration 3 *w*/*w*% kept constant).

Figure 6a–c illustrate the effects of various operating parameters, including temperature, concentration, and flow rate, respectively, on the isoamyl alcohol flux and separation factor. The sparged air flow was kept at 0.25 L/min for the experiments. As the temperature increased from 40 to 80 °C, the flux showed a rise from 0.84 kg/m^2^ h to 1.12 kg/m^2^ h. However, the separation factor displayed a different trend. At lower temperatures, the separation factor increased sharply because of the lower water flux, but in the range of 55–70 °C the increase was more modest. However, at higher temperatures the separation factor increased due to a steep increase in the isoamyl alcohol vapor pressure. This behavior can be attributed to the varying concentrations of water vapor and isoamyl alcohol on the feed side. Meanwhile, the highest flux for the PTFE membrane was found to be 0.43 kg/m^2^ h at a 3% *w*/*w* concentration in the feed. Furthermore, the increase in temperature facilitated the permeation of isoamyl alcohol, which can be attributed to adsorptive transfer. In terms of the concentration effect, higher concentrations were observed to increase both the flux and separation factor due to the generation of a greater number of vapor molecules. Similar trends were observed for the flow rate, as higher flow rates enhanced the chances of active transport. This observation aligns with the contact angle study conducted for the water and isoamyl alcohol system in which membrane selectiveness was observed via preferential sorption.

### 4.3. Prediction of Flux and Separation Factor

The optimization of isoamyl alcohol flux and separation factor for the GOIM was performed based on the Box–Behnken design of the experiment. The Box–Behnken method was chosen because it involves a lower number of experiments to generate a response surface. The process parameters were chosen based on our previous papers. The ranges of the operating variables were chosen as follows: temperature 40–80 °C, feed flow rate of 40–200 mL/min, and a concentration of 1–3% *w*/*w*. This design resulted in 17 experiments, including 5 central points. The flux and separation factor were recorded for all the runs and response-surface plots were generated.

**Figure 7:**
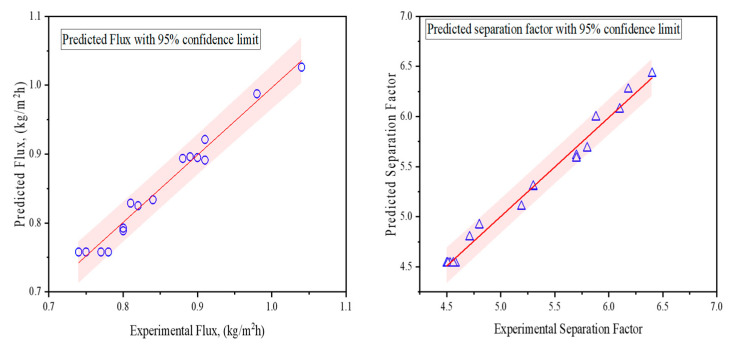
Flux and separation factor prediction for isoamyl alcohol prediction in AS-SGMD using the GOIM membrane.

The goal here was to maximize the flux and separation factor. However, these parameters often tend to be inversely correlated because high flux can lead to a low separation factor. Our objective was to optimize conditions so that a balance between separation factor and flux was obtained. An empirical approach was developed to predict the flux and separation factor. Figure 7 shows the linear regression model for the prediction of the isoamyl alcohol flux and separation factor obtained using Equation (4). The model was developed based on the effect of the operating variables. Both the flux and separation factor showed quadratic effects; the coefficients are listed in Table 1. It is noteworthy that all of the coefficients showed a positive impact for the flux and separation factor. The positive coefficients for the interaction parameters and quadratic effect also justify the curvature effect found in the response surfaces.

The regression equation for the flux and separation factor is.

R = b_0_ + k_1_T + k_2_C + k_3_F + k_4_TC +k_5_TF + k_6_CF + k_7_T^2^ + k_8_C^2^ + k_9_F^2^ (4)

where T is the temperature, C is the concentration, F is the flow rate, and k is the coefficient.

The analysis of variance was performed and the R^2^ values are listed. The R^2^ and predicted R^2^ were similar (difference < 0.20), which is required for the model development. The regression equations are used to determine the optimized operating conditions for the maximum flux and separation factor. The model can be used to predict the performance of the MD within the range of operating variables.

The optimization of the responses was performed based on the overall desirability using the regression equation, keeping the interaction and quadratic terms in consideration. The optimum operating conditions in terms of temperature were 77.8 °C, a flow rate of 195 mL/min, and a concentration of 2.6 *w*/*w*%. The optimized flux within these conditions is 1.041 kg/m^2^ h and a separation factor of 6.30. The overall desirability is 0.97.

**Figure 8:**
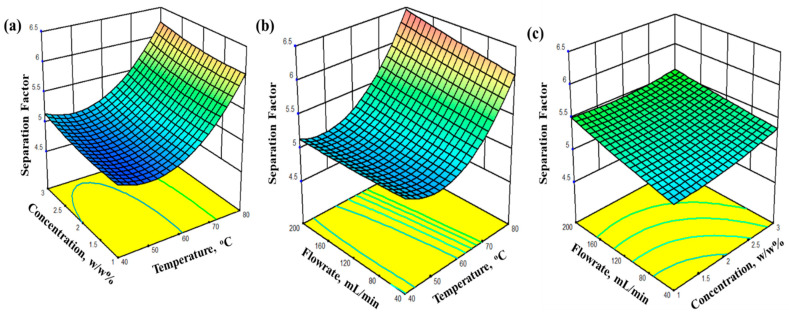
Response-surface plots of the isoamyl alcohol separation factor for the GOIM membrane (**a**) constant flowrate (**b**) constant concentration (**c**) constant temperature

The surface plot helps to predict the response at any combination of operating variables. For the isoamyl alcohol/water system, the response surfaces with contour lines are shown in Figure 8a–c. The response surfaces of the separation factor are plotted against two variables while the third variable is kept constant at the maximum level. The separation factor was strongly affected by the temperature and concentration. The curvature effect in the surface plots arises due to the quadratic dependency on temperature and concentration. A strong interaction was seen for the concentration and temperature pair. Meanwhile, the flow rate data exhibited good separation factors at a high temperature and concentration.

**Figure 9:**
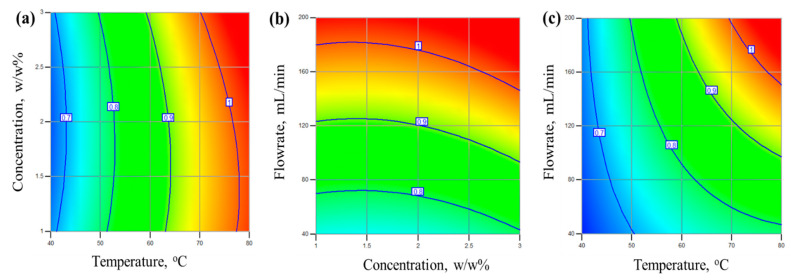
Contour plot of the isoamyl alcohol flux with respect to operating variables in the GOIM membrane (**a**) constant flowrate (**b**) constant temperature (**c**) constant concentration.

The contour plot from the response surfaces for the isoamyl alcohol flux using the GOIM are shown in Figure 9a–c. The contour plot was generated from the quadratic regression equation. The color distribution shows the areas where the maximum flux can be achievable. Here, the orange-red colored area shows the maximum flux region. The interaction and quadratic coefficients are positive for the flux shown in Table 1. Figure 9a shows that the highest flux is achievable with maximum temperature and concentration. Meanwhile, for the flowrate and concentration pair, the flux showed a strong interaction and curvature effect. A similar trend was observed for the visible flow rate and temperature pair. A higher flux of isoamyl alcohol was seen at 70–80 °C and a 2–3% concentration. This is because of the higher isoamyl alcohol vapor pressure at a higher temperature and higher concentration. A large number of molecules can come into contact with the immobilized graphene nanoparticles and pass through the membrane.

### 4.4. Mechanism of Isoamyl Alcohol Enrichment

**Figure 10:**
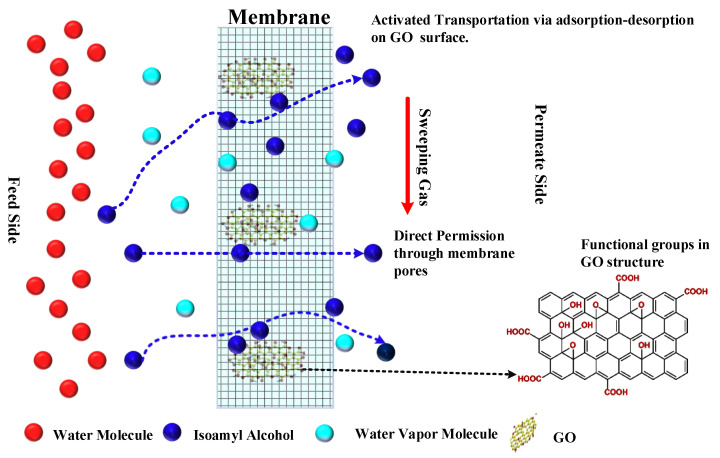
Mechanism of isoamyl alcohol enrichment through the GOIM during membrane distillation.

The primary challenge in membrane distillation (MD) for the separation of isoamyl alcohol is its lower vapor pressure compared to water. At the MD operating temperature, vapors of both water and isoamyl alcohol are generated and traverse through the pores of the membrane. Achieving a high separation factor necessitates the preferential passage of isoamyl alcohol through these membrane pores. The graphene-oxide-modified PTFE membrane (GOIM) offers a solution by exhibiting preferential sorption of isoamyl alcohol at its -OH and -COOH functional sites, followed by rapid desorption facilitated by the temperature gradient. The proposed transport mechanism for isoamyl alcohol permeation through various membranes is visually represented in Figure 10. The introduction of GO onto the membrane surface significantly enhanced the flux of isoamyl alcohol. This enhancement is attributed to the distinctive properties of GO, characterized by a high specific surface area and active sites that facilitate the efficient transport of organic molecules. GO enabled the accelerated transport of isoamyl alcohol molecules through active diffusion via the functional groups. These findings contribute valuable insights into the mechanisms underlying the enhanced performance observed in the GOIM.

## 5. Conclusions

The separation performance of isoamyl alcohol from aqueous solution was evaluated using modified membrane distillation employing air sparging and a graphene oxide immobilized membrane. The GOIM demonstrated improved permeation of isoamyl alcohol through its preferential transport mechanism. Employing the Box–Behnken Method, experiments were systematically designed to investigate the impact of operating temperature, feed concentration, and feed flow rate, employing response-surface methodology and regression modeling. The GOIM achieved an isoamyl alcohol flux of up to 1.17 kg/m^2^, accompanied by a separation factor of 6.7. The flux and separation factor values from the regression models closely matched the experimental data, yielding high R^2^ values of 0.96. Importantly, substantial interaction and curvature effects were revealed regarding the operating variables.

Considering that alcohol for fuels is commonly derived from renewable biomass and agricultural wastes, the imperative of achieving a higher yield for subsequent conversion into more valuable products is highlighted. This study underscores that the utilization of graphene oxide immobilized membrane (GOIM) with air-sparged sweep gas membrane distillation offers a promising approach, resulting in a higher separation factor for isoamyl alcohol and similar compounds. This has significant implications for advancing separation processes within the context of renewable resources.

## Figures and Tables

**Table 1:** Regression coefficients for isoamyl alcohol flux and separation factor prediction.

Coefficient	b_0_	k_1_	k_2_	k_3_	k_4_	k_5_	k_6_	k_7_	k_8_	k_9_
Flux	0.76	0.066	0.031	0.001	0.015	0.052	0.00025	0.081	0.36	0.069
Selectivity	4.54	0.088	0.24	0.090	0.050	0.48	0.15	1.01	0.34	0.22

## Data Availability

The data presented in this study are available on request from the corresponding author.
